# Added value of diffusion-weighted imaging in hepatic tumors and its impact on patient management

**DOI:** 10.1186/s40644-018-0140-1

**Published:** 2018-03-07

**Authors:** Jana Taron, Jonas Johannink, Michael Bitzer, Konstantin Nikolaou, Mike Notohamiprodjo, Rüdiger Hoffmann

**Affiliations:** 1 Department of Diagnostic and Interventional Radiology, University Hospital of Tuebingen, Hoppe-Seyler-Str. 3, 72076 Tuebingen, Germany; 2 Department of Visceral Surgery, University Hospital of Tuebingen, Tuebingen, Germany; 3 Department of Internal Medicine, University Hospital of Tuebingen, Tuebingen, Germany

**Keywords:** Diffusion-weighted imaging, MRI, Additional value, Abdominal imaging, Liver tumors, Oncology

## Abstract

**Background:**

To investigate the added diagnostic value of diffusion-weighted imaging (DWI) of the liver and its impact on therapy decisions in patients with hepatic malignancy.

**Methods:**

Interdisciplinary gastrointestinal tumorboard cases concerning patients with hepatic malignancies discussed between 11/2015 and 06/2016 were included in this retrospective, single-center study. Two radiologists independently reviewed the respective liver MR-examination first without, then with DWI. The readers were blinded regarding number, position and size of hepatic malignancies. Cases in which DWI revealed additional findings concerning the hepatic tumor status as compared to conventional sequences alone were presented to experienced members of the interdisciplinary tumor board. In this retrospective setting changes in treatment decisions based on these additional findings in the DWI sequences were recorded.

**Results:**

A total of 87 patients were included. DWI revealed additional findings in 12 patients (13,8%). These new findings had a direct effect on the therapy in 8 patients (9,2%): In 6 patients (6,9%) the surgical/interventional treatment was adapted (*n* = 5: extended resection, *n* = 1: with transarterial chemoembolization of a single hepatocellular carcinoma only detectable in DWI); 2 patients (2,3%) received systemic therapy (n = 1: neo-adjuvant, n = 1: palliative) based on the additional findings in DWI. In 4 patients (4.6%) additional DWI findings did not affect the therapeutic decision.

**Conclusions:**

DWI is a relevant diagnostic tool in oncologic imaging of the liver. By providing further information regarding tumor load in hepatic malignancies it can lead to a significant change in treatment.

## Background

Over the past two decades diffusion-weighted imaging (DWI) has evolved from being limited to intracranial implementations to an established technique in abdominal magnetic resonance imaging (MRI). Through a series of advances in acquisition techniques, sequence design and hardware, to overcome sensitivity to susceptibility and gross motion, DWI can now be readily integrated into clinical protocols, leading to its widespread use and acceptance [1–3]. Based on the principle of quantifying the degree of free or rather limited Brownian motion of protons [4], DWI inherits the property of detecting diffusion restriction in areas with high cellularity (i.e. malignancies) and cell membrane density. This ability to gather information on changes of diffusion on a cellular level explains its great importance to oncologic imaging [1], where it is used for the detection and characterization of a lesion as well as monitoring and predicting therapeutic response [1, 5–7]. For the management of oncologic patients, particularly with regard to potential surgical options, it is of major importance to reliably detect or rule out metastatic involvement. In abdominal imaging, the liver is of particular interest as it is a common site for metastatic lesions as well as primary malignancies [8, 9].

Several studies have assessed the diagnostic accuracy of DWI for the detection of intrahepatic lesions. However to our knowledge, a study evaluating the impact and added value of diffusion-weighted imaging on therapeutic decision making and, thus, its direct impact on patient care has not yet been performed. As DWI has become an integral part of our daily imaging routine and a reliable diagnostic tool, this subject appears to be of major importance. Thus, aim of our investigation was to quantify the additional information gained through the performance of DWI in oncologic imaging of the liver and evaluate its clinical impact on patient management and therapeutic options.

## Methods

### Patient cohort

This retrospective single-center study was approved by the local institutional review board and written-informed consent was waived. We included all patients with primary hepatic tumors or hepatic metastases, who were referred to our interdisciplinary gastrointestinal tumor board between November 2015 and June 2016. Inclusion criteria was an existing on-site MRI examination (1.5 or 3 T, MAGNETOM Aera/Avanto/Skyra Siemens Healthcare GmbH, Erlangen, Germany) of the abdomen or the liver at the date of tumor board inclusion.

### MR imaging protocol

Institutional standard imaging protocols consisted of the following sequences (acquired in the same order as listed): coronal T2-weighted half acquisition single shot turbo spin echo (HASTE), axial T2-weighted turbo spin echo sequence (TSE) and spectral fat saturation pulse (SPIR) for fat suppression, axial T1 in- and opposed-phase, axial echo planar imaging (EPI) for diffusion-weighted imaging with *b*-values of 0, 400 and 800 s/mm^2^ and dynamic axial T1-weighted volumetric interpolated breath-hold examination (VIBE) sequence with fat saturation using the Dixon technique [10] after intravenous injection of 0.1 mmol of gadobutrol (Gadovist, Bayer HealthCare, Leverkusen, Germany) per kg body weight or 0.025 mmol of gadoxetate disodium (Primovist, Bayer HealthCare) per kg body weight. The intravenous application of contrast medium in patients with highly impaired renal function was spared.

### Image Reading.

Evaluated was the current MRI examination at the date of tumor board inclusion. The MR images were reviewed by two independent and blinded radiologists (R.H. and J.T.; with 6 and 4 year of experience in abdominal MR-imaging). First, the readers reviewed the conventional sequences excluding DWI regarding tumor localization, size and number. Thereafter, reading was repeated using conventional sequences including DWI. Deviations between both reading sessions (with and without DWI) were noted and the corresponding cases were reassessed in a consensus reading.

In a next step, cases with additional findings as agreed upon in the consensus reading were presented (using the available clinical data and liver MRI) to members of the institution’s gastrointestinal tumor board consisting of an abdominal surgeon, a radiologist and a gastrointestinal oncologist (J.J., M.N., M.B.), who were not involved in initial board meeting and, thus, blinded to the actual therapeutic decision. The cases were re-discussed in this simulated setting of the repeated tumor board and hypothetical changes in treatment based on these additional findings in the DWI sequences were recorded and classified as: (a) Change in surgical/interventional procedure, (b) change in systemic treatment, (c) no change in treatment.

Finally, follow-up examinations and clinical reports were examined with respect to the additionally found lesions. If available, these lesions were matched to pathology reports, otherwise appearance and characteristics in the follow-up images served as validation.

Comprehensive data analysis was performed using Microsoft Excel (2007).

## Results

A total of 87 patients (62 male, mean age 70.5 ± 10.5) were included in this study; in 57 patients MRI was performed using gadobutrol, 30 patients received gadoxetate disodium. Patient characteristics were the following: 43 patients presented hepatic metastases (*n* = 26 colorectal carcinomas, *n* = 5 neuroendocrine tumors (NET), *n* = 3 breast carcinomas, *n* = 1 bronchial carcinoma n = 1 melanoma, n = 1 oesophageal carcinoma, *n* = 4 pancreatic carcinoma, n = 1 ovarian carcinoma, n = 1 urothelial carcinoma), 35 patients hepatocellular carcinomas (HCC), 6 patients cholangiocarcinomas (CC) and 3 patients HCC-CC mixed-type carcinoma.

**Fig. 1:**
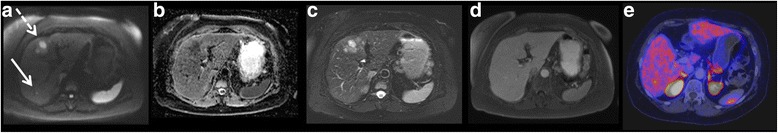
55 year old female patient with hepatic metastases (dashed arrow) of a neuroendocrine tumor. Hepatic lesion (white arrow) was only detected in DWI. The simulated tumorboard decided on change in surgical procedure due to this additionally found lesion. **a**. Diffusion-weighted sequene (b-value 800). **b**. Corresponding ADC map. **c**. T2-weighted sequence. **d**. T1-weighted post-contrast scan. **e**. Corresponding PET-CT of hepatic lesion

**Fig. 2:**
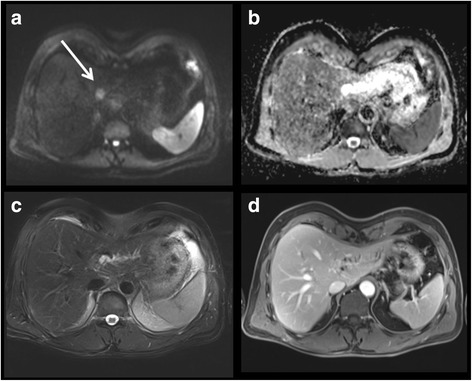
48 year old male patient with hepatic metastases of a pancreatic carcinoma. Additional lesion in Counaud Segment II (white arrow). The simulated tumorboard decided on change in surgical procedure. **a**. Diffusion-weighted image (b-value 800). **b**. Corresponding ADC map. **c**. T2-weighted sequence. **d**. T1-weighted post-contrast scan

**Fig. 3:**
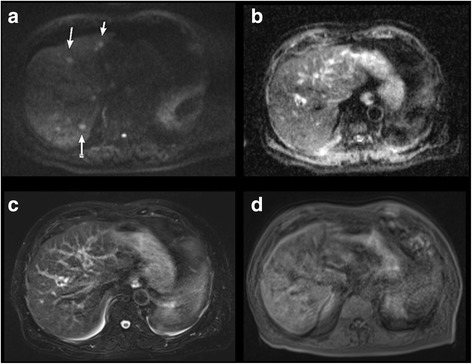
80 year-old male patient with hepatic metastases of a pancreatic carcinoma. Imaging was performed without the intravenous application of contrast material due to highly elevated retention parameters. Motion artifacts due to difficulty in breathing with impairment in image quality. Hepatic lesions were only visible in diffusion-weighted sequences (white arrows). The simulated tumorboard decided on palliative regimen. **a**. Diffusion-weighted image (b-value 800). **b**. Corresponding ADC map. **c**. T2-weighted sequence. **d**. T1-weighted sequence

**Fig. 4:**
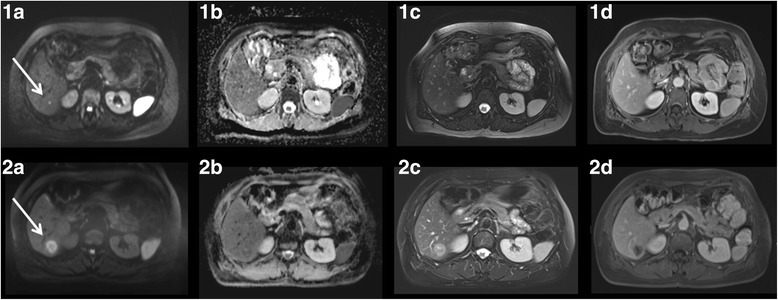
51 year old female patient with hepatic metastases of a neuroendocrine tumor which was only detectable in DWI (white arrow). The simulated tumorboard decided on neoadjuvant chemotherapy. 1) Image series at baseline, 2) Image series after neoadjuvant chemotherapy with progressive disease. **a**. Diffusion-weighted image (b-value 800). **b**. Corresponding ADC map. **c**. T2-weighted sequence. **d**. T1-weighted post-contrast scan

In 12/87 patients (13.8%) DWI revealed additional lesions as compared to reading the conventional MRI protocol alone. In the simulated tumor board setting the members agreed on a change in therapy in 8 of these cases (9.2%). Changes in management were as follows: In 6 patients (6.9%) the surgical treatment or interventional treatment was adapted with an extended resection in 5 cases (*n* = 2 NET, *n* = 1 colorectal carcinoma, n = 1 oesophageal carcinoma, n = 1 pancreatic carcinoma) and a transarterial chemoembolization in one case in which HCC was only identified in DWI; 2 patients (2,3%) received systemic therapy (*n* = 1 neo-adjuvant systemic therapy in a patient with colorectal cancer, n = 1 palliative systemic therapy in a patient with pancreatic cancer). (Figures 1, 2, 3 and 4).

In the other 4 cases (*n* = 4.6%) additional findings did not affect the therapeutic decision due to multiple lesions (detectable in morphological sequences) which already indicated a palliative regimen (*n* = 2 colorectal cancer, n = 1 bronchial carcinoma, n = 1 CC).

Mean follow-up period of the 12 patients with additionally found lesions was 10.5 months [1; 24]. In two cases, the malignant entity of the additional found lesions by DWI could not be definitely confirmed. In both cases (n = 2 colorectal carcinoma) the lesions appeared cystic without progression in long-term follow-up of 17 and 28 months, respectively. A definite differentiation between benign hepatic cyst and cystic residuum was not possible in both cases. The simulated tumor board had decided on ‘no effect on therapy’ based on the additional findings in these two patients due to the presence of multiple intra- and extrahepatic lesions as described above. Regarding the other 10/12 cases, follow-up or histopathology confirmed the malignancy of the additionally found lesions. In these cases 6 patients presented progressive disease (*n* = 1 bronchial carcinoma, n = 1 CC, n = 1 neuroendocrine tumor n = 1 colorectal carcinoma, n = 1 oesophageal carcinoma, n = 1 pancreatic cancer, n = 1 bronchial carcinoma), 2 patients presented regressive disease under chemotherapy (n = 1 pancreatic cancer, n = neuroendocrine tumor), 1 patient presented a relapse of hepatic metastases of colorectal cancer, 1 patient was tumor-free after transarterial chemoembolization of HCC during our follow-up period.

## Discussion

The results of our evaluation show that in about 14% of all patients with hepatic tumor lesions referred to our interdisciplinary tumor board additional suspicious hepatic lesions, which might have otherwise gone undetected, could be identified with diffusion-weighted imaging and, thus, be considered in decisions on individual therapeutic management.

This is especially relevant as the liver is a prevalent location in metastatic disease [9]. Additionally, the number of primary hepatic malignancies is steadily increasing, drawing a focus on HCC and CC- the two most common primary hepatic neoplasms [11–14]. In this context it is crucial to diagnose hepatic involvement for correct patient care in terms of systemic therapy or potential surgical/interventional options.

According to recent literature, the additional information gained by diffusion-weighting is thought to have a direct impact on surgical decisions as well as follow-up after an operational procedure [8, 9], a statement we can now corroborate with our results. While DWI revealed additional lesions in 13.8% of the patients, these findings changed surgical management in 9.2% and or indicated the need for a systemic/palliative therapy in 2.3%.

Previous studies have assessed the role of diffusion-weighted imaging in standard imaging protocols which generally consist of a T2-weighted as well as a T1-weighted in- and opposed phase sequence and a series of contrast enhanced images [8, 15]. In this context, DWI was found to be of higher sensitivity than T2-weighted images in detection of focal liver lesions, and a combination of a diffusion-weighted sequence plus contrast-enhanced series was described to deliver the highest sensitivity for the discovery of suspicious lesions [9, 16]. Colagrande et al. obtained similar results in their recently published study evaluating the value of DWI in contrast-enhanced scans in hepatic metastases of colorectal cancer. They found an improvement on diagnostic accuracy in non-contrast-enhanced examinations due to DWI as well as an increase in specificity in contrast-enhanced images [17].

Metastatic lesions were by far the most common entity in our patient collective and our results match the experience of the above mentioned studies. In our study 11 out of the 12 patients with additional lesions suffered from metastatic diseases. Furthermore, 7 out of the 8 patients with changes in therapy presented with hepatic metastases. This brings us to the assumption that especially in hepatic metastatic disease DWI has an enormous impact on diagnostic imaging from which patients will largely benefit.

Although DWI has already proven to be exceptionally useful in the diagnosis of malignancies [18], its benefits seem to focus on metastases rather than HCC [19]. The detection of HCC in DWI has previously been discussed contradictorily [20, 21]. While larger HCCs usually present typical enhancement characteristics, this behavior alters with decreasing lesion size making it difficult to detect these smaller HCC foci. It is hypothesized that in early tumor development neovascularization might be too faint to be sufficiently recorded by plain or contrast-enhanced images [8, 22]. However, by providing additional information on cell density DWI might be a further puzzle piece on the way to diagnosis [22, 23]. In our study 35 patients presented the diagnosis of a HCC, out of which DWI revealed additional information in one case. Even though there might be restrictions in detecting some cases of HCC in diffusion-weighted images, it needs to be stressed that in this specific example the HCC would have gone undetected without the additional diffusion-weighted sequence.

DWI has also been reported to increase diagnostic sensitivity in the diagnosis of CC [14, 24]. A study performed by Lee et al. revealed that the intensity of diffusion restriction can be used to establish a treatment regimen and improve the outcome of patients with intrahepatic cholangiocarcinoma [25]. Furthermore, DWI may be used in the differential diagnosis of benign strictures and the periductal infiltrating cholangiocarcinoma [14, 26]. Again, DWI in combination with standard abdominal imaging was described to lead to superior diagnosticswhen compared to MRI without an additional diffusion-weighted sequence [26]. With CC-patients being the minority in our collective, a conclusion cannot be made in this context.

Further, with DWI being a non-contrast-enhanced application it plays a special role in the diagnostic performance of patients with impaired renal function [2, 5, 27]. In our experience patients with elevated retention parameters usually present in a critical condition with limited tolerance to lengthy examinations - in these cases DWI is especially relevant as most diffusion-weighted images are performed in free-breathing in a rather short period of time, which naturally plays in favor of limiting artifacts and scan time [2, 3]. The possibility of gaining additional information in an otherwise diagnostically limited image series is a major benefit we noticed in our retrospective evaluation.

There are limitations to this retrospective study. Firstly, imaging was performed with both, scanners of 1.5 and 3 T field strength, which might have affected image quality to a certain extent. Nevertheless, our study protocol was intended to resemble an everyday clinical setting where different scanners (i.e. different magnetic field strengths) are in use and patients are scheduled according to availability. Secondly, for the additionally detected lesions there was no established gold-standard and histological validation was not available for all lesions. In two cases without histopathological evaluation, confirmation of malignancy was not possible on the basis of the follow-up imaging. Therefore, false-positive results of DWI cannot be excluded in these two cases. Also, as above discussed, detection of HCC in diffusion-weighted images can be limited. Further, detailed information on the diagnostic value of DWI in single entities (such as specific metastases, HCC and CC) cannot be made due to small number of cases, so that larger multi-center studies are necessary. Another limitation is the fact that only patients with known hepatic malignancies were included in this evaluation which likely overinflates the rate of patients with additional findings in DWI when compared to a cohort of patients without suspected lesions or with proven extrahepatic malignancies (with a subsequently lower prevalence of liver lesions at all).

## Conclusions

In conclusion, DWI is an important diagnostic tool in oncologic imaging of the liver. In comparison to conventional sequences, DWI reveals further information regarding the tumor load in patients with known hepatic malignancy influencing the therapeutic regimen.

## Abbreviations

CC: Cholangiocarcinoma
DWI: Diffusion-weighted imaging
EPI: Echo planar imaging
HASTE: Half acquisition single shot turbo spin echo
HCC: Hepatocellular carcinoma
MRI: Magnetic resonance imaging
NET: Neuroendocrine tumor
TSW: Turbo spin echo sequence
VIBE: Volumetric interpolated breath-hold examination
